# Preoperative flexion contracture does not impair outcomes or early revision rates following robotic total knee arthroplasty with functional alignment

**DOI:** 10.1002/ksa.12799

**Published:** 2025-07-21

**Authors:** Christos Koutserimpas, Giovan Giuseppe Mazzella, Luca Andriollo, Emanuele Diquattro, Pietro Gregori, Elvire Servien, Cécile Batailler, Sébastien Lustig

**Affiliations:** ^1^ Orthopaedics Surgery and Sports Medicine Department, FIFA Medical Center of Excellence, Croix‐Rousse Hospital Lyon University Hospital, Hospices Civils de Lyon Lyon France; ^2^ 2nd Department of Orthopaedic Surgery ‘Hygeia’ General Hospital of Athens Athens Greece; ^3^ School of Rehabilitation Health Sciences University of Patras Patras Greece; ^4^ Department of Aging, Orthopaedic and Rheumatological Sciences Fondazione Policlinico Universitario Agostino Gemelli IRCCS Rome Italy; ^5^ Sezione di Chirurgia Protesica ad Indirizzo Robotico ‐ Unità di Traumatologia Dello Sport, Ortopedia e Traumatologia Fondazione Poliambulanza Istituto Ospedaliero Brescia Italy; ^6^ SC Ortopedia‐Traumatologia e Chirurgia Protesica e dei Reimpianti di Anca e Ginocchio IRCCS Istituto Ortopedico Rizzoli Bologna Italy; ^7^ Fondazione Policlinico Universitario Campus Bio‐Medico Roma Italy; ^8^ LIBM‐EA 7424, Interuniversity Laboratory of Biology of Mobility, Claude Bernard Lyon 1 University Lyon France; ^9^ IFSTTAR, LBMC UMR_T9406 Univ Lyon, Claude Bernard Lyon 1 University Lyon France

**Keywords:** functional alignment, functional knee positioning, image‐based robotics, robotic knee

## Abstract

**Purpose:**

Preoperative flexion contracture remains a challenging deformity in total knee arthroplasty (TKA). This study aimed to evaluate whether the presence of preoperative flexion contracture influences outcomes and early revision rates following robotic‐assisted TKA performed with functional alignment (FA) principles.

**Methods:**

This retrospective comparative study analysed 190 patients who underwent robotic‐assisted TKA using a computed tomography‐based FA strategy. Patients were grouped based on intraoperative measurement of flexion contracture: ≥10° (study group; 43 patients) and <10° (control group; 147 patients). Clinical outcomes, intraoperative data, and early revision rates were assessed at a minimum 24‐month follow‐up.

**Results:**

The study group exhibited significantly more varus alignment intraoperatively and required greater lateral tibial and posterior medial femoral resections. Preoperative knee flexion was lower in the contracture group (110° vs. 120°, *p* = 0.0018), and postoperative flexion remained slightly reduced (120° vs. 130°, *p* = 0.05). Flexion contracture at follow‐up was 1° in the study group versus 0° in controls (*p* = 0.04). However, no significant differences were observed in Knee Society Scores, Forgotten Joint Score, Kujala score, or early revision rates. All‐cause revision rates were similar (97.67% vs. 98.64%, *p* = 0.66), with a hazard ratio of 1.85 (95% CI: 0.12–27.72). Aseptic survivorship was 100% in the contracture group versus 99.32% in controls (*p* = 0.59).

**Conclusion:**

Patients with preoperative flexion contracture ≥ 10° achieved comparable mid‐term outcomes and early survivorship to those without contracture following robotic‐assisted TKA using FA. These findings support FA as a reliable strategy to manage complex deformities without the need for soft tissue releases.

**Level of Evidence:**

Level III.

**Abbreviations**
BMI: body mass index
CI: confidence interval
CS: cruciate‐substituting
CT: computed tomography
DAIR: debridement, antibiotics, and implant retention
FA: functional alignment
FJS: Forgotten Joint Score
HKA: hip‐knee‐ankle angle
ICC: intraclass correlation coefficient
IQR: interquartile range
KSS: Knee Society Score
MA: mechanical alignment
OA: osteoarthritis
PCL: posterior cruciate ligament
PS: posterior‐stabilised
ROM: range of motion
TEA: transepicondylar axis
TKA: total knee arthroplasty

## INTRODUCTION

The management of preoperative flexion contracture remains a challenge in total knee arthroplasty (TKA) and it may be linked to suboptimal functional outcomes [8, 33]. Posterior soft tissue contracture, particularly involving the posterior capsule and potentially the posterior cruciate ligament (PCL), has been implicated in the development of flexion deformity in osteoarthritic knees, along with the formation of posterior femoral osteophytes in patients with osteoarthritis (OA) [20, 21, 35]. Literature indicates that over one‐third of OA patients undergoing TKA present with knee flexion contracture [33]. This deformity impairs gait, increases energy expenditure, heightens fall risk and accelerates wear on proximal joints, such as the hip and lumbar spine [8]. The inability to restore full knee extension is one of the most significant factors limiting optimal knee function, as a lack of extension leads to functional instability and diminished joint mechanics.

While the accuracy and ideal techniques for measuring laxity are yet to be fully established, robotic technologies have proven to be reliable and safe, while they enable a quantifiable intraoperative evaluation of soft tissue balance, representing a notable advancement compared to previous methods [9, 11, 12, 24]. The functional alignment (FA) or functional knee position philosophy has emerged as a promising solution to address the complexities of knee deformities, focusing on accommodating soft tissue dynamics, bony anatomy and restoration of the anterior compartment aided by a robotic platform [3, 4, 14, 23, 25, 28, 30]. However, the impact of increased preoperative flexion contracture on outcomes in the setting of FA has not yet been evaluated.

The aim of this retrospective comparative study is to evaluate whether the presence of preoperative flexion contracture influences clinical outcomes and early revision rates in patients undergoing robotic‐assisted TKA using FA principles with a minimum follow‐up of two years. The hypothesis was that despite the presence of preoperative flexion contracture, FA in robotic‐assisted TKA would result in comparable outcomes and early survivorship to patients without contracture.

## METHODS

This retrospective comparative study analysed a prospectively maintained database of patients who underwent robotic‐assisted TKA using FA principles from March 2021 to February 2023. Patients were divided into two groups based on the presence or absence of preoperative flexion contracture. The study group included patients with an intraoperatively measured knee flexion contracture ≥10°, while the control group consisted of those with <10°. The intraoperative measurement of flexion contracture was performed using the Mako, Stryker (Mako Surgical Corp.) robotic system. all patients received a cruciate‐substituting (CS) or posterior‐stabilised (PS) fixed‐bearing implant (Triathlon). Both CS and PS have exhibited similar outcomes in robotic TKA with the FA principles [27]. The minimum follow‐up for all included patients was 24 months, allowing for a comprehensive evaluation of clinical and radiological outcomes. This investigation was carried out at a single center, where all surgeries were performed by a specialised team of orthopaedic surgeons. A uniform approach was maintained for perioperative and postoperative care, including anaesthesia, thromboprophylaxis, pain management, and rehabilitation, ensuring consistency across all patients.

Patients were excluded if they underwent TKA using mechanical alignment principles due to pre‐existing soft tissue deficiencies, such as previous fractures, periarticular osteotomies, or significant ligamentous injuries that necessitated deviation from FA. Cases with inadequate follow‐up, defined as less than 24 months, were also excluded. Additionally, patients with incomplete preoperative imaging or intraoperative robotic navigation data were not considered for analysis to ensure methodological consistency.

FA in robotic‐assisted TKA is planned using a computed tomography (CT)‐based system, where femoral and tibial implants are positioned to restore native coronal and sagittal alignments while accounting for bone wear [14, 36]. Preoperatively, bone wear is assessed during preoperative planning using CT‐based robotic software, which allows three‐dimensional visualisation of the femoral and tibial epiphyses [23]. Implant positioning is individualised by evaluating epiphyseal alignment and adjusting resection levels to restore the native joint line while correcting for asymmetrical wear, particularly on the more degenerated condyles. In the coronal plane, tibial alignment is planned within 6° varus to 2° valgus and femoral alignment within 6° valgus to 3° varus. Axial alignment is guided by the transepicondylar axis (TEA) for the femur, and the anteroposterior axis (Akagi line) for the tibia, ensuring optimal rotational positioning and bone coverage. Sagittal positioning is adapted to the patient's tibial posterior slope and implant design [14, 23]. In particular, tibial slope is set between 0°–3° for CS and 0°–1° for PS implants [14, 23, 36]. Intraoperatively, a digital tensioner evaluates flexion and extension gaps, targeting 0–1.5 mm of residual laxity with an additional 1–2 mm of lateral laxity in flexion. Adjustments are made dynamically to optimise joint balance while preserving native soft tissue structures, and final implant positioning is verified through trialling before fixation. The PCL was resected in all patients, including those receiving CS inserts. No other soft tissue releases are performed.

Patient demographics, including age, sex, and body mass index (BMI), were documented. Patients were categorised based on the presence or absence of preoperative flexion contracture (study group: knee flexion contracture ≥ 10°), with radiographic analysis performed preoperatively and postoperatively. Coronal alignment was assessed using the mechanical hip‐knee‐ankle angle (HKA) measured from full‐length standing X‐rays. Furthermore, the posterior tibial slope was measured preoperatively on standardised weight‐bearing lateral knee radiographs. The proximal tibial anatomical axis was defined by drawing two circles within the tibial diaphysis, with their centres connected to form the reference axis. The proximal circle was positioned just below the tibial tubercle, touching anterior, posterior, and superior cortices. The second, distal circle was placed approximately 10 cm further down the diaphysis, as described by Hudek et al. [19]. The posterior slope was then calculated as the angle between this axis and a tangent to the medial tibial plateau. All radiographic measurements were performed using the hospital's PACS system and recorded to the nearest 1°. Interobserver reliability was calculated using all radiographs included in the study, with measurements independently performed by two investigators. Interobserver reliability was excellent, with an intraclass correlation coefficient (ICC) of 0.91 for preoperative HKA, 0.92 for postoperative HKA, and 0.89 for posterior tibial slope.

Intraoperative data on implant positioning were collected using the robotic system which provides output values with a resolution of 0.1°. The system provided real‐time varus/valgus alignment measurements derived from preoperative CT scans. Femoral coronal alignment (varus/valgus) and flexion were measured relative to the mechanical axis of the femur, while tibial coronal alignment (varus/valgus) and posterior slope were referenced to the mechanical axis of the tibia. Femoral component rotation was assessed using the surgical TEA, while tibial component rotation was aligned to the Akagi line.

The thickness of the bone resections, including medial and lateral tibial cuts, as well as distal medial, distal lateral, posterior medial, and posterior lateral femoral cuts, was recorded directly from the intraoperative data provided by the robotic system which provides output values with a resolution of 0.1 mm. Functional outcomes were assessed preoperatively and at the final follow‐up using the Knee Society Scores (KSS) for knee function and overall performance, while range of motion (ROM) was evaluated based on active knee flexion. The Forgotten Joint Score (FJS) and the Kujala score were measured at the final follow‐up. Survival analysis of the implants was performed to compare early revision rates between the groups, incorporating all‐cause and aseptic revisions.

### Statistical analyses

The distribution of continuous variables was assessed using the Kolmogorov–Smirnov test to determine normality. Based on the data characteristics, comparisons between groups were conducted using either the independent *t*‐test for normally distributed variables or the Mann–Whitney *U*‐test for non‐parametric data. Categorical variables, including complication rates, were analysed using the chi‐square test. Statistical significance was set at a *p*‐value of less than 0.05. Implant survival was examined through Kaplan–Meier analysis to evaluate differences in revision rates between groups. All statistical analyses were performed using MedCalc software (version 22.021). This investigation followed an ‘all‐comers’ design, including consecutive patients who met the inclusion criteria during the study period. As a retrospective cohort study, no a priori power analysis was performed.

## RESULTS

A total of 190 patients were included in the study, with a median age of 70 years (interquartile range [IQR] 63–75) and a median BMI of 28.3 kg/m² (IQR 25.5–31.7). The cohort comprised 103 females, representing 54.21% of the total population. The median follow‐up period was 36 months (IQR 28–38).

The study group (43 patients) had a median preoperative flexion contracture of 11°, while the control group (147 patients) had 3°. CS inserts were used in 13.95% of patients in the study group and 26.53% in the control group (*p* = 0.09). Intraoperatively, the study group exhibited significantly greater median varus alignment as measured by the robotic system (8° vs. 6°, *p* = 0.03) and had reduced median preoperative active knee flexion compared to the control group (110° vs. 120°, *p* = 0.0018). No significant differences were observed between groups regarding age, BMI, KSS, HKA or tibial posterior slope (Table 1).

**Table 1:** Preoperative characteristics of the study group (flexion contracture ≥ 10°) and control group (<10°) are summarised, including demographics, clinical scores and radiographic parameters.

		Study group (*N* = 43)	Control (*N* = 147)	*p*‐Value
Demographics	Age (years)	72 (IQR 63.3–75.8)	70 (IQR 63.3–74.8)	0.34
	Female gender			
	BMI (kg/m^2^)	28.3 (IQR 25.3–32)	28.3 (IQR 25.6–31.6)	0.64
Preoperative clinical evaluation	KSS‐knee	63.2 (SD = 15.3)	65.2 (SD = 12)	0.37
	KSS‐function	70 (IQR 55–80)	70 (IQR 50–80)	0.25
	Active knee flexion (clinical evaluation)	110° (IQR 110–120)	120° (IQR 111.3–130)	**0.0018**
Preoperative radiological evaluation	HKA	174° (IQR 170–177)	173° (IQR 171–176)	0.56
	Tibial posterior slope	7° (IQR 4.3–9)	7° (IQR 6–9)	0.65
Intraoperative assessment with the Robotic system	Alignment (varus)	8° (IQR 5–10)	6° (IQR 4–8.8)	**0.03**
	Flexion contracture	11° (IQR 10–14)	3° (IQR 1–6)	**<0.0001**

*Note*: Statistically significant differences are indicated in bold. Based on the data characteristics, comparisons between groups were conducted using either the independent *t*‐test for normally distributed variables (KSS‐knee) or the Mann–Whitney *U*‐test for non‐parametric data (all the other parameters expect KSS‐knee).

Abbreviations: BMI, body mass index; HKA, hip‐knee angle; IQR, interquartile range; KSS, Knee Society Score; *N*, number.

Intraoperative analysis of implant positioning showed that median femoral component valgus alignment was significantly lower in the study group compared to the control group (0° [IQR −1 to 1.3] vs. 1° [IQR 0–2], *p* = 0.02) (Table 2).

**Table 2:** Femoral and tibial implant positioning during the robotic‐assisted total knee arthroplasty under the functional alignment principles.

		Study group (*N* = 43)	Control (*N* = 147)	*p*‐Value
Tibial implant positioning (degrees with reference to the tibial mechanical axis)	Varus	4° (IQR 2.6–5)	3.5° (IQR 2–4.5)	0.18
	Posterior slope	1° (IQR 0–1)	0° (IQR 0–1)	0.32
Femoral implant positioning (degrees with reference to the femoral mechanical axis)	External rotation (reference TEA)	0° (IQR −0.9 to 1.1)	0.2° (IQR −1.3 to 1.5)	0.93
	Valgus	0° (IQR −1 to 1.3)	1° (IQR 0–2)	**0.02**
	Flexion	6.2° (IQR 3.2–8.6)	7° (IQR 5.5–9)	0.05

*Note*: Study group: knee flexion contracture ≥ 10° and the control group: Knee flexion contracture < 10°. The statistically significant values are depicted in bold. Based on the data characteristics, comparisons between groups were conducted using the Mann–Whitney *U*‐test for non‐parametric data.

Abbreviations: IQR, interquartile range; TEA, transepicondylar axis.

Analysis of bone resection thickness revealed that the study group demonstrated a greater lateral tibial resection (median 8.5 vs. 8 mm) and posterior medial femoral resection (median 9 mm vs. 9 mm) compared to the control group (Table 3).

**Table 3:** Thickness of the cuts between the study and control group.

		Study group (*N* = 43)	Control (*N* = 147)	*p*‐Value
Tibial cuts (mm)	Medial	7.5 (IQR 6.38–8.5)	8 (IQR 7–8)	0.72
	Lateral	8.5 (IQR 8–9)	8 (IQR 7.5–8.5)	**0.015**
Femoral cuts (mm)	Distal medial	9 (IQR 9–9.5)	9 (IQR 8.5–9.5)	0.19
	Distal lateral	8.5 (IQR 7.5–9)	8.5 (IQR 7.5–9)	0.87
	Posterior medial	9 (IQR 9–9.5)	9 (IQR 8.5–9.5)	**0.028**
	Posterior lateral	8 (IQR 7.5–9)	8.5 (IQR 7.5–9)	0.62

*Note*: Study group: knee flexion contracture ≥ 10° and the control group: knee flexion contracture < 10°. The statistically significant values are depicted in bold. Based on the data characteristics, comparisons between groups were conducted using the Mann–Whitney *U*‐test for non‐parametric data.

Abbreviation: IQR, interquartile range.

Postoperative evaluation showed that active knee flexion remained significantly lower in the study group (120° vs. 130°, *p* = 0.05). Residual flexion contracture was also higher in the study group at the final follow‐up (1° vs. 0°, *p* = 0.04). No significant differences were observed between groups in postoperative KSS, FJS, Kujala score or HKA (Table 4).

**Table 4:** Postoperative evaluation of the clinical, radiographic parameters between the two groups.

		Study group (*N* = 43)	Control (*N* = 147)	*p*‐Value
Postoperative clinical evaluation	KSS‐knee	91 (IQR 90–100)	95 (IQR 90–100)	0.61
	KSS‐function	90 (IQR 90–100)	93 (IQR 90–100)	0.61
	FJS	80 (IQR 56–90)	82 (IQR 58–92)	0.8
	Kujala Score	92 (IQR 82–98)	93 (IQR 84–100)	0.81
	Active knee flexion (degrees)	120 (IQR 120–130)	130 (IQR 120–130)	0.05
Postoperative coronal alignment evaluation Assessment from the robotic system	HKA (degrees)	178° (IQR 175–179)	178° (IQR 176–179.13)	0.23
	Postoperative Mako Alignment (varus‐degrees)	4° (IQR 1–6)	3° (IQR 2–5)	0.2
	Flexion contracture (degrees)	1° (IQR 0–2)	0° (IQR −1 to 2)	**0.04**

*Note*: Study group: knee flexion contracture ≥ 10° and the control group: knee flexion contracture < 10°. The statistically significant values are depicted in bold. Based on the data characteristics, comparisons between groups were conducted using the Mann–Whitney *U*‐test for non‐parametric data.

Abbreviations: FJS, Forgotten Joint Score; HKA, hip‐knee angle; IQR, interquartile range; KSS, Knee Society Score; *N*, number.

**Figure 1:**
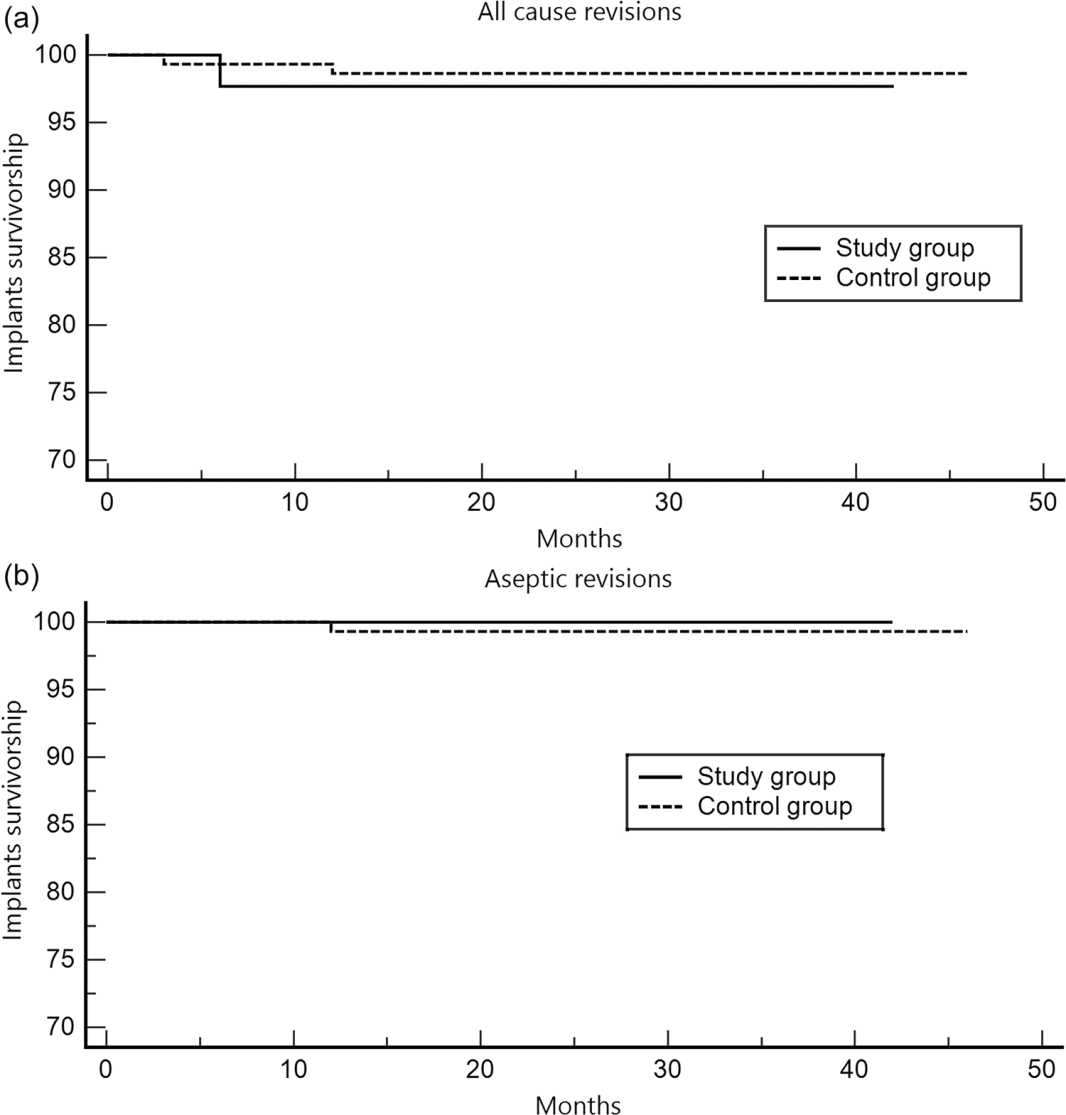
(a) Kaplan–Meier survival analysis for all‐cause revision, comparing the study and control group. The survival rate was 97.67% in the study group and 98.64% in the control group (*p* = 0.66). The hazard ratio for all‐cause revision was 1.85 (95% CI: 0.12–27.72) for the study group and 0.54 (95% CI: 0.03–8.09) for the control group. Study group: knee flexion contracture ≥ 10° and the control group: knee flexion contracture < 10°. (b) Kaplan–Meier survival analysis for aseptic loosening, comparing the study and control groups. The survival rate was 100% in the study group and 99.32% in the control group (*p* = 0.59). Study group: knee flexion contracture ≥ 10° and the control group: knee flexion contracture < 10°. CI, confidence interval.

Survival analysis showed no significant difference in all‐cause revision rates between the study and control groups, with survival rates of 97.67% and 98.64%, respectively (*p* = 0.66). One case of debridement, antibiotics, and implant retention (DAIR) was recorded in each group, while an additional femoral component revision was observed in the control group. The hazard ratio for all‐cause revision was 1.85 (95% confidence interval [CI]: 0.12–27.72) for the study group and 0.54 (95% CI: 0.03–8.09) for the control group. Regarding aseptic survivorship, no revisions were recorded in the study group, while the control group had a single case of aseptic failure, leading to a survival rate of 100% in the study group and 99.32% in the control group (*p* = 0.59) (Figure 1a,b).

## DISCUSSION

This study evaluated the influence of preoperative flexion contracture on outcomes and early revision rates in patients undergoing robotic‐assisted TKA guided by FA principles. The main finding of this investigation is that the presence of a flexion contracture ≥10° did not adversely affect postoperative clinical scores or revision rates when compared to patients without contracture. Despite presenting with more varus alignment intraoperatively, reduced preoperative flexion, and requiring greater tibial and femoral resections, patients in the flexion contracture group achieved comparable functional outcomes, including KSS‐knee and ‐function, FJS, and Kujala score at mid‐term follow‐up. These results suggest that the FA philosophy, supported by robotic assistance, allows for individualised soft tissue and bony adjustments that accommodate preoperative contractures without compromising clinical efficacy or implant longevity.

Patients with preoperative flexion contracture (≥10°) exhibited greater intraoperative varus alignment, suggesting a possible link between sagittal and coronal plane deformities, likely due to asymmetrical degeneration and soft tissue contractures in advanced OA. Although this association has not been thoroughly investigated, some studies have reported that knees with flexion contracture tend to present with varus alignment [31]. For example, Han et al. reported a mean preoperative HKA of 4° varus in patients with flexion contracture, suggesting that these deformities may frequently coexist [17]. This relationship remains poorly characterised in the literature. Notably, discrepancies between intraoperative robotic alignment and preoperative radiographic HKA likely reflect differences in imaging conditions; non‐weight‐bearing CT in supine position versus weight‐bearing full‐length radiographs, which can affect limb alignment [15]. Patients with flexion contracture showed reduced active knee flexion, consistent with literature indicating that contracture and limited flexion often coexist as features of generalised joint stiffness [2, 7, 8]. Biomechanically, this can be attributed to chronic soft tissue adaptations, including posterior capsular contracture, quadriceps shortening, and periarticular fibrosis, all of which contribute to limited mobility in both extension and flexion [8]. In this study, the only soft tissue release performed was PCL resection, applied uniformly for both CS and PS implants as part of the surgical technique. No additional releases were necessary, even in severe contractures, as balance was achieved through bony resections under the FA principles. PS implants were more common in the control group (26.53% vs. 13.95%), though this was not statistically significant (*p* = 0.09). A recent study on FA similarly reported no difference in outcomes between CS and PS inserts, supporting their combined analysis [27].

Differences in implant positioning and bone resections in the flexion contracture group reflect the three‐dimensional adjustments required to achieve balance without soft tissue release. Increased lateral tibial resection likely compensated for greater varus alignment and asymmetric wear, while the thicker posterior medial femoral cut addressed sagittal stiffness from posterior capsular contracture to restore extension without altering the joint line. Though these resections address different planes, they function together as complementary adjustments guided by robotic gap assessment. Furthermore, although the present study did not aim to replicate native anatomical alignment, some deviation from population‐based anatomical averages, such as femoral coronal alignment or rotational orientation, was observed [6, 18]. These deviations reflect the individualised, morphology‐driven planning principles of FA, which prioritises flexion‐extension balance, and implant congruence rather than adherence to fixed anatomical norms [5, 14, 22, 23, 26, 28]. As such, coronal femoral alignment and axial rotation were guided by the patient‐specific epiphyseal geometry and soft tissue envelope, within established safe zones, rather than by population means derived from healthy knees [14, 23, 36].

Although the flexion contracture group had slightly lower final flexion and higher residual contracture, the absolute outcomes; 120° flexion and 1° contracture, remain satisfactory given their more limited preoperative status. These results suggest that meaningful functional gains are achievable, even if full restoration is limited by preoperative pathology [1, 10, 32, 37].

Only a few studies in the literature have specifically examined outcomes TKA in patients with flexion contracture, particularly in the context of mechanically aligned TKA, and the results have been divergent. Lu et al. reported acceptable outcomes in a small cohort of patients with severe flexion contracture (mean 78°), though the follow‐up data were limited and the surgical challenges were substantial [29]. Guan et al., in a series of 97 cases, showed that although patients with > 20° contracture had worse preoperative function, their postoperative outcomes were comparable to those with milder deformities [16]. Fernandez et al., in a large retrospective study including 480 patients with preoperative flexion contracture and 2152 controls, reported that clinical outcomes after TKA did not significantly differ between groups, with a mean follow‐up of 3.3 years [13]. Conversely, Ritter et al., in a large retrospective review of over 5600 mechanically aligned TKAs, found that patients with ≥ 6° of preoperative flexion contracture were more likely to have residual contracture postoperatively and demonstrated significantly worse pain, knee and function scores [33]. These studies suggest that under mechanical alignment principles, flexion contracture may predispose patients to suboptimal postoperative motion and persistent symptoms. In contrast, the current study, using the FA approach, demonstrated that patients with ≥ 10° of preoperative flexion contracture achieved outcomes and early implant survivorship equivalent to those without contracture. These favourable results, achieved without soft tissue releases, emphasise the potential of FA to accommodate complex preoperative deformities through personalised bony corrections and intraoperative balancing, rather than relying on standard alignment targets or aggressive releases that may compromise soft tissue integrity. Traditional management of flexion contracture often involves posterior capsular release, removal of posterior osteophytes, or additional distal femoral bone resection to regain full extension; however, such techniques, while effective, can risk altering joint kinematics and inducing mid‐flexion instability [34]. This highlights the advantage of FA as a dynamic and adaptable strategy in modern TKA, particularly in challenging anatomical scenarios, by achieving balance and alignment through precise, individualised bone resections rather than generalised corrective manoeuvres.

This study has some limitations. First, its retrospective design introduces the possibility of selection and information bias, despite the use of a prospectively maintained database. Second, the investigation was conducted at a single center, which may limit generalisability; however, this also ensured a standardised surgical technique, consistent perioperative protocols, and uniform application of FA principles. Furthermore, the follow‐up duration, while adequate for assessing early clinical outcomes and implant survivorship, does not capture long‐term durability or late complications. Additionally, the study was not designed or powered for a formal equivalence or non‐inferiority analysis. While no statistically significant differences were found between groups, these results should not be interpreted as proof of equivalence but rather as evidence that outcomes in patients with flexion contracture were not inferior within the observed clinical context. Moreover, implant selection (CS vs. PS) was not randomised. Although the distribution of insert types did not significantly differ between groups, no stratified analysis was performed due to the relatively small number of CS cases, which may have limited the ability to detect implant‐specific effects on outcomes. Nevertheless, to our knowledge, this is the first study to specifically evaluate the impact of preoperative flexion contracture in the setting of FA, providing novel insight into how this modern alignment strategy can address complex deformities without compromising outcomes.

## CONCLUSIONS

This study demonstrated that preoperative flexion contracture ≥ 10° did not negatively impact mid‐term clinical outcomes or implant survivorship in patients who underwent robotic‐assisted TKA using FA principles. Despite requiring tailored bony corrections, patients with flexion contracture achieved comparable results to those without contracture, without the need for soft tissue releases. These findings highlighted the capacity of FA to effectively manage complex deformities through individualised, three‐dimensional surgical planning.

## AUTHOR CONTRIBUTIONS

*Conceptualisation*: Christos Koutserimpas, Giovan Giuseppe Mazzella, Cécile Batailler and Sébastien Lustig. *Methodology*: Giovan Giuseppe Mazzella, Emanuele Diquattro, Elvire Servien, Cécile Batailler and Sébastien Lustig. *Formal analysis and investigation*: Christos Koutserimpas, Giovan Giuseppe Mazzella, Emanuele Diquattro, Pietro Gregori and Luca Andriollo. *Writing—original draft preparation*: Christos Koutserimpas, Giovan Giuseppe Mazzella, Emanuele Diquattro, Pietro Gregori and Luca Andriollo. *Writing—review and editing*: Elvire Servien, Cécile Batailler, Sébastien Lustig; Supervision: Cécile Batailler, Sébastien Lustig.

## CONFLICTS OF INTEREST STATEMENT

Christos Koutserimpas, Giovan Giuseppe Mazzella, Luca Andriollo, Emanuele Diquattro and Pietro Gregori have nothing to declare. Elvire Servien: Consultant for Smith and Nephew. Cécile Batailler: Consultant for Smith and Nephew and Stryker. Sébastien Lustig: Consultant for Heraeus, Stryker, Depuy Synthes, Smith and Nephew. Institutional research support to Lepine and Amplitude.

## ETHICS STATEMENT

This study was conducted in accordance with the ethical guidelines set by the institutional and national research committees, adhering to the principles of the 1964 Declaration of Helsinki and its subsequent amendments. Data collection and analysis complied with the MR004 Reference Methodology established by the Commission Nationale de l'Informatique et des Libertés (Reference No. 2229975V0). Given the retrospective nature of the study and the use of anonymized data, formal patient consent was waived in accordance with institutional regulations.

## Data Availability

The data that support the findings of this study are available from the corresponding author, upon reasonable request.
